# The relationship between parenting style and bystander’s promotion of cyberbullying among college students: the mediating effect of neuroticism and moral disengagement

**DOI:** 10.3389/fpsyg.2025.1622523

**Published:** 2025-08-01

**Authors:** Bing Wang, Chang Liu

**Affiliations:** ^1^School of Educational Science, Shenyang Normal University, Shenyang, China; ^2^Social Economy and Management, Woosuk University, Wanju-Gun, Republic of Korea

**Keywords:** parenting style, cyberbullying, neuroticism, mediating effect, college students

## Abstract

To explore the relationship between parenting style and bystander’s promotion of cyberbullying among Chinese college students, as well as the mediating effect of neuroticism and moral disengagement. A total of 495 college students were selected as participants in this study. The short form Egna Minnen av. Barndoms Uppfostran for Chinese, the revised Eysenck Personality Questionnaire-Short Scale for Chinese, Moral Disengagement Scale and Cyberbullying Bystanders Behaviors Questionnaire were used to conduct the test. The results showed that: (1) rejection was positively correlated with neuroticism, moral disengagement and bystander’s promotion of cyberbullying; Emotional warmth was negatively correlated with neuroticism, moral disengagement and bystander’s promotion of cyberbullying. Neuroticism and moral disengagement were positively correlated with bystander’s promotion of cyberbullying. (2) The mediating effect analysis showed that the direct effect of parenting style (rejection and emotional warmth) on the bystander’s promotion of cyberbullying was significant. In the relationship between parenting style (rejection and emotional warmth) and bystander’s promotion of cyberbullying, the mediating effect of neuroticism and moral disengagement and the chain mediating effect of neuroticism and moral disengagement were significant.

## 1 Introduction

With the widespread integration of the internet into daily life, cyberbullying has emerged as a critical societal issue. As an extension of traditional school bullying, cyberbullying often inflicts more severe psychological harm due to its anonymity and pervasive nature (Du, 2013). While existing research predominantly focuses on cyberbullies and victims, the role of bystanders—individuals who witness cyberbullying—remains underexplored. Cyberbullying bystanders are defined as individuals who witness online harassment incidents (Huang et al., 2019; Sobol et al., 2025). While this definition originates from research in the Chinese context, it aligns with cross-cultural conceptualizations of bystander roles (e.g., Panumaporn et al., 2020; Pfetsch, 2017), emphasizing observation without direct involvement. Established research classifies bystander behaviors into two categories: positive bystander behavior and negative bystander behavior, with the latter encompassing behaviors that reinforce the cyberbullying and assist the cyberbullying (Zhao et al., 2023). Although most bystanders choose inaction, which is cross-culturally prevalent (Huang et al., 2019), 35.6% actively support perpetrators (Wu, 2009), exacerbating victims’ psychological trauma and fostering antisocial tendencies (Pfetsch, 2017). Bystander’s promotion of cyberbullying (BPC) refers to actions such as liking, sharing aggressive content, or posting inflammatory comments that directly or indirectly support cyberbullies’ harmful actions (Teng, 2015). Notably, older adolescents are more likely to join perpetrators (Panumaporn et al., 2020). Critically, bystander behavior is shaped by developmental antecedents beyond immediate situational factors. Rooted in ecological systems theory (Bronfenbrenner, 1979), familial influences—particularly parenting styles—establish enduring cognitive and emotional templates that guide responses to online social conflicts, underscoring the urgency of investigating factors influencing bystander behavior among college students to mitigate harm and uphold digital ethics.

This study holds dual significance in both theoretical and practical aspects. Theoretically, it (1) extends the application of the ecological systems theory to digital contexts by revealing the predictive role of parenting styles (rejection and emotional warmth) as distal environmental variables on BPC; (2) proposes a chain mediation mechanism of “neuroticism → moral disengagement” to elucidate the psychological pathway through which bystanders actively reinforce harm rather than passively avoid it, thereby addressing the path dependency on perpetrator mechanisms in existing research; (3) integrates the General Aggression Model with the theory of moral disengagement, establishing neuroticism as a developmental antecedent (rather than a moderating variable) of moral disengagement, thus resolving theoretical disputes.

Practically, the findings of this study will provide a scientific basis for (1) the development of family-based parent–child communication training programs (e.g., reducing rejection behaviors and enhancing emotional responsiveness); (2) the design of campus intervention projects focusing on emotional regulation for individuals with neuroticism and cognitive restructuring of moral disengagement; (3) the formulation of policy frameworks emphasizing digital citizenship responsibility. Particularly in the context of China’s collectivist culture, where family values significantly shape social cognition (Hu and Xiong, 2024), this discovery holds greater targeted value.

### 1.1 Parenting styles and BPC

Rooted in ecological systems theory, family environments—particularly parenting styles—profoundly shape individual development. Parenting styles reflect caregivers’ educational philosophies and behavioral tendencies in daily interactions (Jiang et al., 2021). Empirical studies consistently link parenting styles to children’s behavioral outcomes (Chen, 2024; Zhou et al., 2024a). For instance, warm, supportive, and authoritative parenting serves as a protective factor against cyberbullying for both victims and perpetrators (Cerezo et al., 2018). Conversely, rejecting parenting (e.g., criticism, neglect) correlates positively with cyberbullying (Zhi et al., 2018). Effective parent–child communication fostered by positive parenting reduces online aggression (Appel et al., 2014), while hostile parenting perpetuates conflict and normalizes violent attitudes (Buelga et al., 2017; Wang et al., 2020). Rejecting parents often neglect supervision and guidance, increasing adolescents’ susceptibility to engaging in or supporting cyberbullying (Lu, 2021). However, BPC—though functionally aligned with perpetration—represents a distinct behavioral phenomenon with unique psychological underpinnings. Unlike perpetrators who initiate aggression, bystanders reinforce harm through passive endorsement (e.g., sharing/liking content). This normalization of violence relies more heavily on moral disengagement (justifying inaction/complicity) than the active hostility driving perpetration (Wang et al., 2016). Reducing bystander support is clinically prioritized as it collectively amplifies harm (Pfetsch, 2017), yet parenting’s role in this specific behavior remains unexamined. Critically, while parenting styles predict perpetration, their impact on bystanders likely operates through distinct pathways (e.g., neuroticism-driven passivity vs. perpetrator-focused callousness; Barlett and Anderson, 2012). Thus, we hypothesize:

*H1:* Positive parenting (emotional warmth) negatively predicts BPC, whereas negative parenting (rejection) positively predicts BPC.

### 1.2 The mediating role of neuroticism

In addition to subtly influencing children’s behavior, parenting styles directly shape their personality traits. Positive parenting—characterized by respect, responsiveness to children’s needs, and emotional warmth—fosters autonomy, security, and prosocial personality development. Conversely, negative parenting (e.g., rejection, hostility, overcontrol) serves as a risk factor for maladaptive traits such as neuroticism (Ferencz et al., 2022; Zeng, 2012). Neuroticism, a core personality trait, reflects heightened sensitivity to perceived threats and emotional instability. Individuals with high neuroticism exhibit anxiety, mood swings, and poor self-regulation. Research demonstrates that rejecting parenting exacerbates neurotic tendencies by undermining parent–child attachment and amplifying insecurity (Liu and Yuan, 2019). For instance, Wang et al. (2006) found that parental rejection positively correlates with adolescent neuroticism, while emotional warmth inversely relates to it. Children raised in hostile environments often develop perfectionism, distrust, and emotional volatility, further predisposing them to maladaptive behaviors.

Neuroticism also significantly positively predicts cyberbullying-related behaviors (Xu et al., 2024). Neuroticism also significantly predicts cyberbullying-related behaviors. Its hallmark features—emotional instability, impulsivity, and poor self-regulation—hinder prosocial bystander actions (Erreygers et al., 2016). Critically, however, neuroticism is not merely associated with passivity; it actively predisposes individuals toward promoting cyberbullying through distinct affective-cognitive pathways. First, high neuroticism amplifies threat sensitivity and defensive aggression (Zhu and Xia, 2021), leading bystanders to perceive alignment with aggressors as a strategy to avoid becoming targets themselves (Barlett and Anderson, 2012). Second, neurotic individuals experience intensified negative affect (e.g., anxiety, irritability) when witnessing conflict, which may be maladaptively regulated through displaced aggression (e.g., liking/sharing harmful content) to alleviate distress. Third, their impulsivity and susceptibility to peer influence increase the likelihood of conforming to aggressive group norms to gain social acceptance (Zhou et al., 2024a; Zhou et al., 2024b). Empirical evidence confirms this directional link: Zhou et al. (2018) found neuroticism positively predicted bystander promotion but not defending behaviors, while Erreygers et al. (2016) observed that neurotic impulsivity drove active reinforcement of bullying among adolescents. Thus, neuroticism transcends passive avoidance by fostering motivational states (self-protection, affect regulation, social conformity) that actively enable complicity with perpetrators. Based on the established link between neuroticism and BPC—driven by threat sensitivity, negative affect regulation, and peer conformity—we hypothesize:

*H2:* Parenting styles influence BPC through the mediating role of neuroticism.

### 1.3 The mediating role of moral disengagement

The General Aggression Model (GAM) posits that environmental and individual factors jointly shape aggressive behaviors through cognitive pathways (Kowalski et al., 2014). Personality traits, such as neuroticism, may distort social cognition (e.g., justifying aggression) to increase cyberbullying propensity. Moral disengagement—a cognitive mechanism enabling individuals to rationalize unethical acts while avoiding guilt—plays a pivotal role here (Bandura, 1990). Recent studies reveal nuanced mechanisms linking neuroticism to moral disengagement, presenting two distinct relationships:

1. Neuroticism as a moderator: Zhou et al. (2024a) and Zhou et al. (2024b) demonstrated that neuroticism moderates the indirect pathway from deviant peer affiliation to cyberbullying via moral disengagement, with effects stronger in high-neuroticism individuals.
2. Neuroticism as a direct antecedent: Meta-analytic evidence confirms neuroticism’s robust direct effect on moral disengagement (Luo and Bussey, 2023), independent of contextual triggers.

Despite this apparent tension, we hypothesize a serial mediation model (parenting → neuroticism → moral disengagement → bystander promotion) for three theoretically grounded reasons: first, developmental primacy supports temporal sequencing. Neuroticism emerges as a stable trait shaped early by parenting (Liu and Yuan, 2019), whereas moral disengagement represents malleable cognitive scripts activated later in response to situational cues (Bandura et al., 1996). This temporal precedence positions neuroticism as an antecedent to moral disengagement in the causal chain (Luo and Bussey, 2023). Second, the affective-cognitive pathway in GAM necessitates serial mediation. Neuroticism generates intense negative affect (e.g., anxiety during cyberbullying exposure), which motivates moral disengagement as a distress-regulation strategy (Bandura et al., 1996). For example, neurotic bystanders may deploy victim blaming (“They provoked it”) to alleviate their discomfort, transforming affective vulnerability into cognitive justification (Zhou et al., 2024a; Zhou et al., 2024b). Third, bystander contexts demand active cognitive restructuring. Passive avoidance (e.g., outsider behavior) requires minimal justification, but actively reinforcing bullying (e.g., sharing content) necessitates moral disengagement to override guilt (Teng, 2015). Neuroticism fuels this process by intensifying the affective drive for self-exoneration—a sequential mechanism empirically validated in cyberbullying perpetration (Luo and Bussey, 2023) but untested for bystanders. Thus, while neuroticism may moderate moral disengagement’s effects in specific contexts, our model prioritizes its role as a developmental precursor and affective catalyst for moral disengagement in bystander scenarios.

According to Bandura’s theory (1996), moral standards typically inhibit harmful behavior, but disengagement strategies (e.g., blaming victims, diffusing responsibility) override these restraints. Critically, parenting styles shape the development of these strategies: emotional warmth (measured here) aligns with authoritative parenting’s core dimension of responsiveness and consistent guidance, which fosters moral internalization and reduces moral disengagement (Liu and Lu, 2013). Conversely, parental rejection (our negative parenting measure) shares conceptual overlap with permissive/authoritarian parenting through its lack of accountability modeling and emotional neglect, actively normalizing disengagement mechanisms (Buelga et al., 2017; Hu and Xiong, 2024).

Regarding bystander behavior, anonymity in online environments facilitates moral disengagement by reducing perceived accountability (Wang et al., 2016). However, moral disengagement is not merely a co-result of anonymity; it acts as a direct cognitive enabler of harmful behavior. Empirical evidence confirms that even when controlling for anonymity, moral disengagement directly predicts bystander promotion of cyberbullying (Wang and Ngai, 2020), as it cognitively reframes harmful actions as acceptable (e.g., “Sharing this post is not my fault—the victim deserved it”). This causal link is further supported by longitudinal studies showing moral disengagement precedes and fuels cyber-aggression (Luo and Bussey, 2023). Thus, we propose:

*H3:* Parenting styles exert indirect effects on BPC through moral disengagement.

*H4:* Parenting styles exert indirect effects on BPC through a chain mediation pathway: neuroticism → moral disengagement.

Neuroticism and moral disengagement were selected as sequential mediators due to their unique theoretical and mechanistic significance in explaining bystander promotion of cyberbullying (BPC). Neuroticism serves as the critical affective bridge linking parenting styles to online behavior: unlike broad traits (e.g., agreeableness), it is highly sensitive to familial influences (Liu and Yuan, 2019) and generates threat reactivity and negative affect (e.g., anxiety during conflicts) that motivate complicity with aggressors (Barlett and Anderson, 2012; Zhou et al., 2024a; Zhou et al., 2024b). Moral disengagement is the essential cognitive enabler for BPC, as it actively overrides moral inhibitions through justification strategies (e.g., victim blaming)—a mechanism distinct from empathy deficits or normative influences (Bandura et al., 1996; Wang et al., 2016). Crucially, their sequential order reflects a causal affective-cognitive progression: neuroticism’s distress precipitates moral disengagement as a distress-regulation tool (e.g., “They deserved it” reduces anxiety), which then licenses harmful actions (Luo and Bussey, 2023; Zhou et al., 2024a; Zhou et al., 2024b). This chain is amplified in online contexts, where anonymity intensifies neurotic individuals’ reliance on moral disengagement to alleviate discomfort while avoiding guilt (Chu et al., 2024). Thus, the neuroticism → moral disengagement pathway uniquely elucidates why bystanders actively promote harm rather than merely withdraw—a mechanism empirically validated for perpetration but novel for bystander complicity.

In summary, while parenting styles correlate with cyberbullying perpetration/victimization (Cerezo et al., 2018), their impact on bystander choices (e.g., promoting vs. intervening) is rarely examined, especially in collectivist cultures where familial values heavily shape social cognition (Hu and Xiong, 2024). College students represent a high-risk group for bystander complicity due to increased online exposure and evolving autonomy from parents (Panumaporn et al., 2020), yet parental influences during this transitional phase remain overlooked. No prior study has tested whether parenting affects bystander behavior through sequential mediators (neuroticism → moral disengagement), a pathway theoretically grounded in the GAM (Anderson and Bushman, 2002) but unexamined empirically.

This study addresses these gaps by investigating how parental rejection/emotional warmth predicts BPC via neuroticism and moral disengagement. Establishing these pathways provides actionable insights for family-based interventions targeting bystander accountability.

## 2 Materials and methods

### 2.1 Participants

A stratified cluster sampling method was used to randomly select the combined classes of grade one to grade four in two universities in Liaoning Province, and the test was conducted during self-study time. The study design was approved by the Research Ethics Committee of Shenyang Normal University (No. SNU-2023-27). The participants filled in an informed consent form to participate in the study. Five hundred and fifty questionnaires were distributed, 532 questionnaires were collected, and 37 invalid questionnaires were excluded. Finally, 495 valid samples were obtained, with an effective rate of 93.05%. The age of the participants ranged from 17 to 24 years old (*M* = 19.69, *SD* = 1.408). There were 283 males (57.2%) and 212 females (42.8%), including 124 freshmen (25.05%); 151 sophomore students, accounting for 30.51%; 136 junior students, accounting for 27.47%; And 84 senior students, accounting for 16.97%. The number of households in urban was 174 (35.2%); 321 students (64.8%) had rural household registration. In terms of family structure, there were 91 individuals from multigenerational households, accounting for 18.4%; 352 individuals from nuclear families, making up 71.1%; 32 individuals from divorced families, representing 6.5%; 13 individuals from single-parent families due to death of one parent, accounting for 2.6%; 1 orphan, representing 0.2%; and 6 individuals from other family situations, making up 1.2%.

An *a priori* power analysis (G*Power 3.1) indicated a minimum sample of *N* = 420 to detect small-to-medium effects (*β* ≥ 0.20, power = 0.95, *α* = 0.05) in our SEM model. Accounting for cluster sampling and anticipated attrition, we recruited 550 participants. After excluding invalid responses (*n* = 37), the final sample (*N* = 495) exceeded the target, achieving 99% power for detecting small effects (*f*^2^ = 0.05) and maintaining a 29:1 cases-to-parameters ratio, ensuring robust parameter estimation (Jiang et al., 2021; Schreiber et al., 2006).

### 2.2 Measures

#### 2.2.1 Short form Egna Minnen av. Barndoms Uppfostran

The Chinese version of the short-form Egna Minnen av. Barndoms Uppfostran, revised by Jiang et al. (2010), was utilized. This questionnaire comprises 23 items across three dimensions: Rejection (7 items, e.g., “My parents often scolded me for no reason.”), Emotional Warmth (8 items, e.g., “My parents praise me.”), and Overprotection (6 items, e.g., “My parents require me to explain to them what I have been doing outside once I return home.”). Responses were recorded on a 4-point Likert scale, ranging from 1 (“Never”) to 4 (“Always”). The mean score for each subscale was calculated and used in subsequent analyses. The internal consistency coefficients (Cronbach’s *α*) for the subscales were 0.864, 0.785, and 0.701, respectively.

#### 2.2.2 The revised Eysenck personality questionnaire-short scale for Chinese

The Chinese version of the Eysenck Personality Questionnaire-Short Scale (EPQ-RSC), revised by Qian et al. (2000), was employed. This 48-item instrument assesses four dimensions: Neuroticism, Extraversion, Psychoticism, and Social Desirability. Participants responded with “Yes” or “No” to each item. For instance, one item for neuroticism is “I often feel anxious.” The current study utilized the Neuroticism subscale (12 items), which demonstrated good internal consistency (Cronbach’s *α* = 0.508). A mean score was computed, with higher scores indicating greater neuroticism.

#### 2.2.3 Moral disengagement scale

The Moral Disengagement Scale, originally developed by Bandura (1990) and adapted into Chinese by Yang et al. (2015), was administered. The scale comprises 32 items across eight dimensions (4 items each): Diffusion of Responsibility (e.g., “Telling a small lie is not a big deal because it will not really hurt anyone.”), Advantageous Comparison (e.g., “A child should not be punished for the mistakes made by the group he or she belongs to. “), Distortion of Consequences (e.g., “Some people deserve to be treated like animals. “), Dehumanization (e.g., “Compared to hitting someone, damaging some property is not a serious matter.”), Displacement of Responsibility (e.g., “It is acceptable to fight to protect a friend. “), Euphemistic Labeling (e.g., “A child who merely instigates others to violate discipline should not be blamed because others have followed the instigation.”), Attribution of Blame (e.g., “If students cause trouble at school, it’s the teacher’s fault. “), and Moral Justification (e.g., “Using a friend’s bicycle without their permission is just considered ‘borrowing’.”). Participants rated items on a 5-point Likert scale (1 = “Strongly Disagree” to 5 = “Strongly Agree”). The overall moral disengagement score was derived from the mean of all 32 items, with higher scores indicating greater moral disengagement. The subscales exhibited Cronbach’s *α* values ranging from 0.700 to 0.865.

#### 2.2.4 Cyberbullying bystander’s behaviors questionnaire

The Cyberbullying Bystander Behavior Questionnaire, developed by Teng (2015), includes 20 items divided into three subscales: promotion of cyberbullying behavior (7 items, e.g., “I would help the perpetrator forward and spread the incident.”), defending behavior (9 items, e.g., “I would advise people I know not to get involved in the perpetrator’s actions.”), and outsider behavior (4 items, e.g., “I will not help either side.”). Responses were recorded on a 7-point Likert scale (1 = “Completely Disagree” to 7 = “Completely Agree”), with higher scores reflecting stronger behavioral tendencies. The current study focused on the Promotion of Bullying Behavior subscale, which showed excellent reliability (α = 0.956). The mean score of the 7-item promotion of cyberbullying behavior subscale was used as the primary outcome variable.

### 2.3 Procedure

For the online data collection, participants were recruited through student affairs administrators of respective faculties. They were provided with a QR code linked to the survey hosted on a secure online platform. The survey was designed to be mobile-friendly and accessible across various devices. Participants were informed about the purpose of the study and the voluntary nature of their participation. They were required to complete an informed consent form before proceeding to the questionnaire. To ensure a controlled and consistent environment, participants were tested collectively in classrooms. Research assistants were present to answer any questions and ensure that participants understood the instructions. The entire process took approximately 30 min to complete.

### 2.4 Data processing

Data analysis was conducted using SPSS 26.0 for correlation analysis, reliability analysis, and exploratory factor analysis (EFA). Structural equation modeling (SEM) was performed with Mplus 8.0 to examine the hypothesized mediation pathways. To test the mediating effects of neuroticism and moral disengagement between parenting styles and BPC, a bias-corrected nonparametric percentile bootstrap method (1,000 resamples) was applied. All variables were standardized prior to analysis to ensure comparability of coefficients and mitigate scaling effects.

## 3 Results

### 3.1 Common method bias test

To assess potential common method bias, an EFA was conducted following the procedure outlined by Zhou and Long (2004). The results revealed 13 factors with eigenvalues greater than 1. The first common factor accounted for 28.097% of the total variance, which is below the 40% threshold, indicating no severe common method bias in the dataset.

### 3.2 Correlation analysis among variables

A correlation analysis was conducted to examine the relationships among parenting styles (rejection and emotional warmth), neuroticism, moral disengagement, and BPC. The results (see Table 1) revealed that rejection was significantly positively correlated with neuroticism, moral disengagement, and BPC, while emotional warmth showed significant negative correlations with these variables. Additionally, both neuroticism and moral disengagement were significantly positively associated with BPC, further highlighting the interconnected roles of personality traits and cognitive mechanisms in shaping bystander behavior.

**Table 1:** Descriptive statistics and correlations between variables (*n* = 495).

	*M*	*SD*	1	2	3	4	5	6
1. Rejection	1.46	0.48	1					
2. Emotional warmth	2.83	0.57	0.42^**^	1				
3. Over protect	2.00	0.49	0.58^**^	−0.06	1			
4. Neuroticism	0.21	0.15	0.32^**^	−0.32^**^	0.12^**^	1		
5. MD	1.83	0.62	0.40^**^	−0.29^**^	0.24^**^	0.31^**^	1	
6. BSC	1.54	1.15	0.19^**^	−0.15^**^	0.08	0.25^**^	0.40^**^	1

^**^ *p* < 0.01. MD: Moral disengagement; BSC: bystander’s promotion of cyberbullying.

### 3.3 Mediation effect analysis

**Figure 1:**
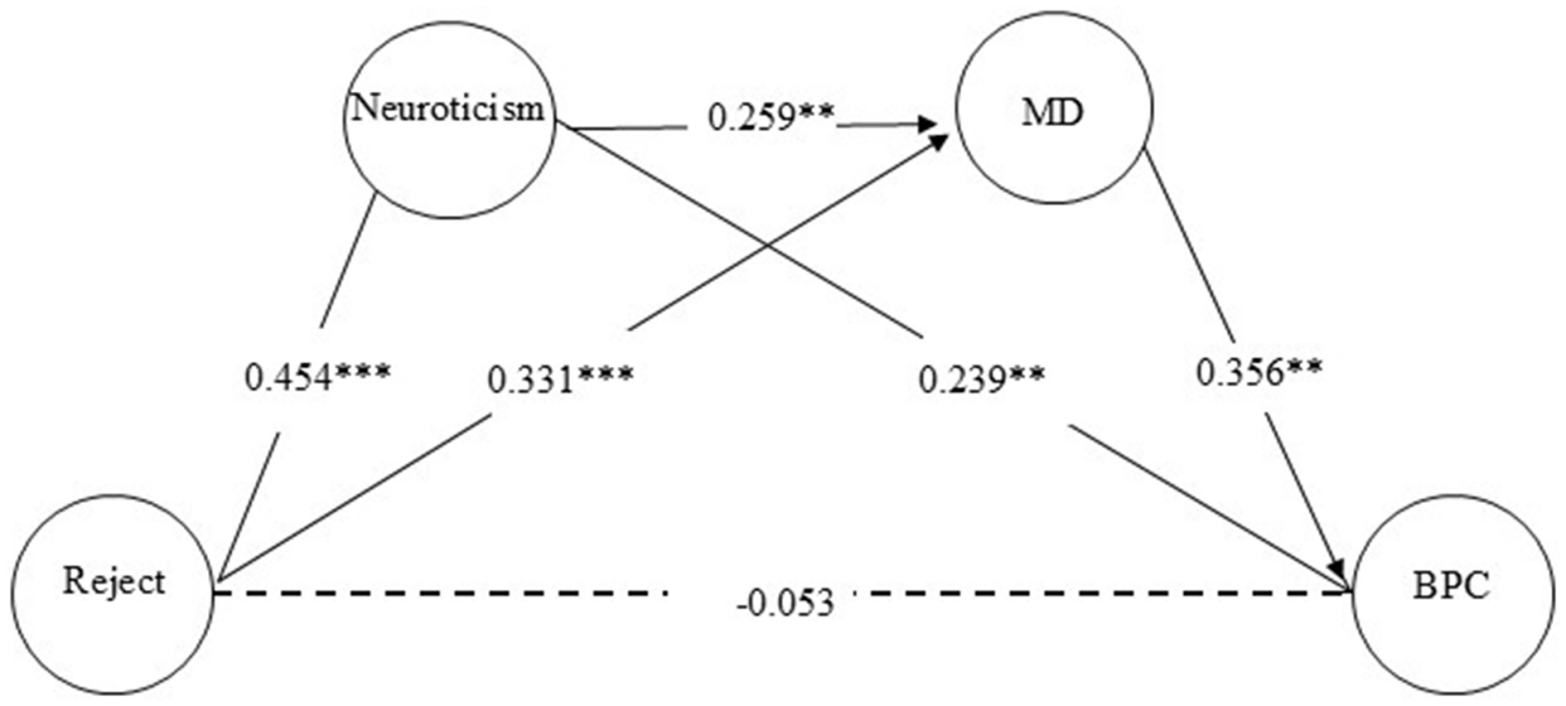
Model of mediating effects of rejection on promoting bullying behavior. ^**^p < 0.01, ^***^p < 0.001. MD: moral disengagement. BPC: bystander’s promotion of cyberbullying.

**Figure 2:**
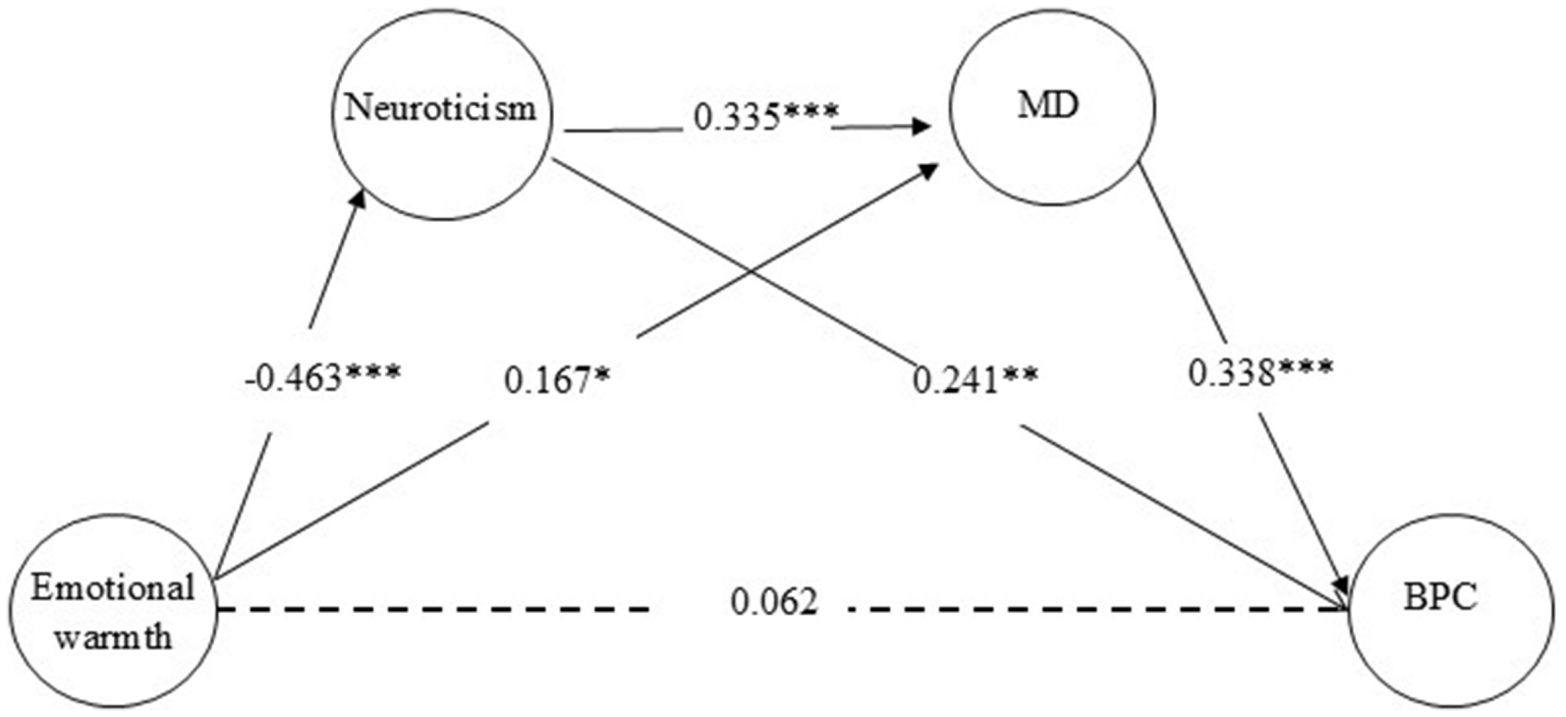
Mediating model of the effect of emotional warmth on promoting bullying behavior. ^*^p < 0.05, ^**^p < 0.01, ^***^p < 0.001. MD: moral disengagement. BPC: bystander’s promotion of cyberbullying.

To analyze the mediating effects, two total effect models were first established: Model 1 examined the total effect of rejection on BPC, while Model 2 assessed the total effect of emotional warmth on BPC. The results demonstrated significant total effects for both rejection (*β* = 0.215, *p* < 0.001) and emotional warmth (*β* = −0.158, *p* < 0.01). This result supports Hypothesis 1. Subsequently, two mediation models were constructed: Model 1 tested the pathway rejection → neuroticism → moral disengagement → BPC, and Model 2 explored emotional warmth → neuroticism → moral disengagement → BPC. Structural equation modeling (SEM) analysis (Figures 1, 2) confirmed acceptable model fit indices for both total and mediation models (see Table 2). To evaluate the significance of mediation pathways, a bias-corrected bootstrap method with 1,000 resamples was employed.

**Table 2:** Fit index of mediation model.

Model	χ^2^	*df*	CFI	TLI	SRMR	RMSEA
Total effect Model 1	340.918^***^	76	0.950	0.940	0.032	0.084
Total effect Model 2	376.327^***^	89	0.951	0.942	0.036	0.081
Mediation effect Model 1	671.750^***^	296	0.961	0.956	0.032	0.055
Mediation effect Model 2	756.538^***^	293	0.957	0.953	0.037	0.057

^***^p < 0.001.

The 95% confidence intervals (CIs) for path coefficients, presented in Table 3, revealed that both mediation models encompassed three distinct effects: (1) independent mediation by neuroticism, supporting Hypothesis 2, (2) independent mediation by moral disengagement, supporting Hypothesis 3, and (3) chain mediation through neuroticism → moral disengagement, supporting Hypothesis 4. These results underscore the dual roles of neuroticism and moral disengagement as both standalone and sequential mediators in the relationship between parenting styles and BPC, emphasizing the complex interplay of psychological mechanisms in shaping behavioral outcomes.

**Table 3:** Bootstrap 95% confidence intervals for the path of mediating effects.

Mediation path	Effect	95% confidence interval	
		Lower limit	Upper limit
Rejection → Neuroticism → BSC	0.108	0.052	0.203
Rejection → Moral disengagement → BSC	0.118	0.071	0.198
Rejection → Neuroticism → Moral disengagement → BSC	0.042	0.021	0.078
Emotional warmth → Neuroticism → BSC	−0.112	−0.428	−0.089
Emotional warmth → moral disengagement → BSC	−0.056	−0.237	0.013
Emotional warmth → neuroticism → moral disengagement → BSC	−0.052	−0.201	−0.053

BSC: bystander’s promotion of cyberbullying.

## 4 Discussion

### 4.1 Relationship between parenting styles and BPC

The results confirmed Hypothesis 1, demonstrating that parental rejection was positively predictive with BPCo, while emotional warmth exhibited a negative predictive with BPC. These findings align with ecological systems theory, indicating that the foundational family environment shapes cognitive and emotional templates that influence responses to online social conflicts like witnessing cyberbullying. Individuals raised in rejecting environments (characterized by criticism, coldness, and neglect) frequently experience chronic feelings of fear, anger, loneliness, and insecurity (Chen et al., 2020). This persistent negative emotional state can foster resentment and hostility toward others. Furthermore, rejecting parenting often fails to model or teach prosocial values, empathy, and effective conflict resolution, instead implicitly normalizing disrespect and aggression as interaction styles (Buelga et al., 2017; Wang et al., 2020). Crucially, the lack of parental warmth and support weakens the parent–child bond, diminishing the child’s motivation to internalize parental moral standards (Bandura, 1990), thereby reducing internal barriers against supporting harmful behaviors. The resulting insecurity and negative affectivity heighten sensitivity to peer influence and increase the likelihood of seeking belonging or reducing personal anxiety by aligning with aggressors, even passively through promotion behaviors like liking or sharing harmful content. Engaging in or supporting bullying may also serve as a maladaptive outlet for their own accumulated negative emotions or as a way to gain perceived status within a hostile online context.

Conversely, emotionally warm parenting (characterized by responsiveness, affection, and support) fosters psychological safety, secure attachment, and healthy self-esteem (Lu, 2021). This supportive environment actively cultivates empathy and perspective-taking skills, enabling individuals to better understand and share the distress of cyberbullying victims (Krishnashree et al., 2022), making the endorsement of harm to others less tolerable. Warm parents typically model and reinforce prosocial behavior, moral reasoning, and responsibility, providing guidance on navigating social dilemmas. Children in such positive relationships are more likely to internalize these values due to the stronger, more positive bond (Liu and Lu, 2013; Hu and Xiong, 2024), building a stronger internal moral compass. Consequently, individuals raised with warmth develop a greater sense of personal responsibility and agency in social situations, making them less likely to passively endorse harm and more inclined toward prosocial actions, or at least avoidance of complicity.

Notably, overprotection showed no significant association with BPC. This non-significant finding suggests distinct pathways for different negative parenting dimensions compared to rejection. It may stem from overprotected individuals developing passive, dependent tendencies and reduced autonomy. They might lack the confidence to actively intervene but also lack the initiative or assertiveness to actively promote bullying. Furthermore, overprotection can sometimes correlate with lower levels of empathy development if it shields children from understanding others’ struggles or taking responsibility (Xu et al., 2008). In the context of cyberbullying bystanderism, this combination of passivity, dependency, and potentially blunted empathy may manifest primarily as outsider behavior (inaction) rather than active promotion. They may avoid involvement altogether rather than actively choose to support the aggressor.

A critical insight from the structural equation modeling (SEM) is that although the total effect model shows that rejection and emotional warmth have significant predictive effects on BPC, SEM revealed no significant *direct* effects of these parenting styles on promotion behavior. This strongly suggests that the associations between parental rejection/warmth and BPC operate *entirely* through the mediating variables investigated in this study – namely, neuroticism and moral disengagement. Parenting styles primarily shape underlying personality traits (neuroticism) and cognitive moral mechanisms (disengagement), which in turn directly influence the behavioral choice to promote cyberbullying when witnessing it.

### 4.2 Mediating roles of neuroticism and moral disengagement

The findings robustly support the mediating roles of both neuroticism and moral disengagement in the link between parenting styles and BPC. Neuroticism mediated the relationship between parenting styles (rejection/emotional warmth) and BPC. This aligns with the GAM, which posits that stable personality traits like neuroticism influence aggressive behavior by shaping an individual’s internal state—particularly their affective and arousal responses. Parenting serves as a key developmental antecedent to neuroticism: Rejecting parenting, characterized by criticism and emotional unavailability, actively exacerbates feelings of insecurity, hypersensitivity, and emotional dysregulation (Zeng, 2012), thereby fostering higher levels of neuroticism. Conversely, emotionally warm parenting, through its nurturing and supportive nature, enhances emotional regulation capacities and buffers against anxiety, leading to lower neuroticism. Highly neurotic individuals, burdened by emotional instability, exhibit distinct cognitive-affective patterns in conflict situations like witnessing cyberbullying: They possess a heightened attentional bias toward potential threats and negative social cues (Zhu and Xia, 2021), often interpreting ambiguous online interactions as hostile. This, coupled with poor impulse control and heightened negative affect (e.g., anxiety, distress), makes them less likely to engage in the emotionally demanding act of defending a victim. Instead, their self-focused distress and avoidance tendencies (Erreygers et al., 2016) may manifest as passive complicity or even active promotion, as aligning with the aggressor might be perceived as a way to reduce their own anxiety or avoid becoming a target themselves. In contrast, the lower neuroticism fostered by emotional warmth facilitates greater empathy and perspective-taking, reducing the propensity to support harm.

Moral disengagement also mediated the parenting-bystander relationship. Rooted in Bandura’s social cognitive theory, moral disengagement represents a set of cognitive mechanisms that allow individuals to selectively deactivate their internal moral self-sanctions, thereby enabling engagement in harmful conduct without experiencing guilt or self-condemnation. Parenting styles critically influence the development of these cognitive mechanisms. Rejecting parenting environments model behaviors such as blame-shifting, responsibility avoidance, and a lack of accountability (Buelga et al., 2017). Children raised in such environments are less likely to develop strong internalized moral standards or learn to take responsibility for the consequences of actions (including inaction), making them more susceptible to employing moral disengagement strategies later in life. Conversely, emotionally warm parenting cultivates a sense of responsibility, moral reasoning, and empathy (Hu and Xiong, 2024). Parents who provide consistent guidance and model prosocial behavior help children internalize moral standards, making it harder for them to cognitively justify harmful acts. The unique context of cyberspace further amplifies the role of moral disengagement: The anonymity, reduced cues about the victim’s suffering, and diffusion of responsibility inherent in online environments (Fang and Wang, 2020; Yang et al., 2015) significantly weaken moral constraints. This makes it easier for bystanders, regardless of upbringing, to employ moral disengagement tactics (e.g., dehumanizing the victim, displacing responsibility to the bully or the platform, minimizing consequences) to rationalize their passive observation or active promotion of cyberbullying, thereby overriding any latent inhibitions.

Most crucially, a significant chain mediation effect was observed, where parenting styles influenced BPC sequentially through neuroticism first, and then moral disengagement. This finding illuminates a complex psychological pathway linking early familial experiences to specific online bystander behavior. Neurotic individuals, experiencing heightened anxiety and emotional vulnerability when confronted with ambiguous or hostile online situations like cyberbullying, are particularly motivated to alleviate their distress. Moral disengagement provides a readily accessible cognitive toolkit for achieving this emotional relief. By employing cognitive distortions such as victim blaming (“They brought it on themselves”), advantageous comparison (“It’s not as bad as what others do”), or diffusion of responsibility (“It’s not my place to intervene”), the neurotic bystander can redefine the harmful event in a way that reduces its perceived wrongfulness and their own personal responsibility (Bandura et al., 1996; Mateus Francisco et al., 2024; Zhou et al., 2024a; Zhou et al., 2024b). This cognitive restructuring effectively suppresses feelings of guilt or empathy that might otherwise arise, allowing the individual to passively endorse or actively promote the bullying (e.g., by liking, sharing, or adding inflammatory comments) without significant internal conflict (Wang et al., 2024). For instance, a neurotic bystander, feeling anxious about potential social exclusion if they do not side with the aggressor, might share derogatory content. Using moral disengagement (e.g., “The victim is annoying anyway” or “Everyone else is doing it”), they justify this action, thereby reducing their anxiety and facilitating the BPC. This sequential process—where heightened negative affect (neuroticism) drives the use of cognitive justification strategies (moral disengagement) to enable harmful inaction or action—is fully consistent with the GAM. GAM explicitly incorporates the interplay between personological factors (like neuroticism) and situational inputs (like witnessing cyberbullying), which interact to influence internal states (cognitions and affect), ultimately leading to behavioral outcomes such as BPC. Here, neuroticism interacts with the online context to weaken moral self-regulation via increased reliance on moral disengagement.

### 4.3 Limitations and future directions

This study has limitations. First, the sample was restricted to college students; future research should include vocational students. Second, only neuroticism was examined; incorporating traits like agreeableness or empathy could refine the psychological mechanism. Third, the cross-sectional design limits causal inferences; longitudinal studies using cross-lagged models are needed. Fourth, although this study utilizes a Chinese sample and references locally developed scales (e.g., EPQ-RSC), the theoretical framework—particularly the GAM and Bandura’s moral disengagement theory—is universally applicable. The mediating pathways (e.g., parenting styles → neuroticism → moral disengagement → bystander’s behavior) reflect psychological mechanisms transcending cultural boundaries, as evidenced by similar findings in Western contexts (e.g., Luo and Bussey, 2023; Zhou et al., 2018). However, cultural factors may modulate effect sizes. For instance, collectivist values in China might amplify the impact of parental rejection on moral disengagement due to heightened familial interdependence (Hu and Xiong, 2024). Future cross-cultural comparisons should validate whether the magnitude of these effects varies across societies. Fifth, although our dimensional approach (positive/negative parenting) is theoretically and empirically justified, collapsing nuanced styles may obscure subtle effects. Future studies should use categorical parenting typologies to test whether authoritative parenting (high warmth + high control) uniquely buffers against BPC compared to supportive parenting (high warmth + low control).

## 5 Conclusion

(1) Parenting styles (rejection and emotional warmth) significantly predict BPC among college students. (2) Neuroticism and moral disengagement act as chain mediators in this relationship, illustrating how familial environments shape personality and cognitive mechanisms to influence bystander behavior. These findings underscore the need for family-centered interventions to mitigate cyberbullying risks.

## Data Availability

The original contributions presented in the study are included in the article/supplementary material, further inquiries can be directed to the corresponding author.
